# Closed Genome Sequence of Noninvasive *Streptococcus pyogenes* M/*emm*3 Strain STAB902

**DOI:** 10.1128/genomeA.00792-14

**Published:** 2014-08-28

**Authors:** Nicolas Soriano, Pascal Vincent, Séverine Moullec, Alexandra Meygret, Vincent Lagente, Samer Kayal, Ahmad Faili

**Affiliations:** a Laboratoire de Microbiologie et Immunologie, Faculté de Médecine et de Pharmacie, Université Rennes 1, Rennes, France; b Service de Bactériologie et Hygiène Hospitalière, CHU Pontchaillou, Rennes, France; c IGDR-UMR 6290-CNRS, Université Rennes 1, Rennes, France; d UMR991-INSERM, Université de Rennes 1, Rennes, France

## Abstract

We report a closed genome sequence of group A *Streptococcus* genotype *emm*3 (GAS M/*emm*3) strain STAB902, isolated from a superficial pyodermatitis. The genome is composed of 1,892,124 bp, 6 integrated prophages, and has 1,858 identified coding sequences (CDSs). It has been fitted with the two available invasive GAS M/*emm*3 strains.

## GENOME ANNOUNCEMENT

*Streptococcus pyogenes* or GAS may be asymptomatically carried in the throat of 5 to 15% of normal individuals and is also an important human pathogen that causes a wide variety of infectious diseases. The bacterial wall-anchored M protein, which is encoded by the *emm* gene, is an important virulence factor and is also an epidemiological marker that is used worldwide to characterize GAS isolates (1, 2). Strains with the M/*emm*3 type are more often associated with invasive infections such as necrotizing fasciitis or streptococcal toxic shock syndrome, and have a higher case fatality rate than strains of most other *emm* genotypes (3, 4). The complete genomes of two invasive GAS M/*emm*3 strains are available (5, 6). Aiming to decipher molecular mechanisms involved in invasiveness by comparing GAS M/*emm*3 strains, we sequenced and annotated the whole genome of an M/*emm*3 GAS strain isolated from a non-invasive superficial cutaneous infection, henceforth named STAB902.

A STAB902 strain grown in Todd-Hewitt medium supplemented with 0.2% yeast extract (THY) and DNA for sequencing was extracted and purified using the phenol-chlorophorm technique. Genomic DNA was sequenced using HiSeq 2000 technology (Illumina, Inc., San Diego, CA) and the paired-end library was built using the MGX facility of the CNRS in Montpellier, France. There are a total of 35,623,128 high-quality reads giving an average of 1,964-fold coverage of the genome, which was assembled using CLC Genomics Workbench v6 software (http://www.clcbio.com). The resulting assembly consisted of 90 contigs, which were oriented and connected based on the two previously sequenced GAS M/*emm*3 strains (SSI-1 and MGAS 315) (5, 6). In comparison with these two reference genomes, which both show large genomic rearrangements (5), the final chromosomal organization of the STAB902 strain is identical to that of the SSI-1 strain. After reassembling, 9 gaps persisted within the repeated zones (4 rRNAs, 3 tRNAs, and 2 hypothetical phage proteins), and were filled according to the nucleotide sequences of the SSI-1 and MGAS 315 strains. Two assembly gaps separated by 278 bp were localized within the *sclB* gene (collagen-like surface protein) (7). Therefore, the *sclB* sequence was determined by PCR and sequencing. This allows the complete closure of the chromosome of the STAB902 strain. Genome annotation was performed in parallel by using the RAST server (8) and NCBI-PGAP (http://ncbi.nlm.nih.gov/genome/annotation_prok). Prophages were identified using the PHAge search tool (PHAST) (9).

Finally the strain STAB902 harbored a single circular genome of 1,892,124 bp, with a G+C-content of 38.4%. We identified 1,858 coding sequences (CDSs), 60 tRNAs genes, 15 rRNAs genes, and 6 intact integrated prophages. The multilocus sequence type (10) is determined to be ST406. In comparison with SSI-1 and MGAS 315 genomes (5, 6), almost all of the genes of known virulence factors (proteinases, superantigen, proteins adhesion, and gene regulator) are present. In addition, genomic comparison evidenced in the strain STAB902 in-frame deletions of *sclB* and *graB* (11) genes encoding two surface proteins anchored to the bacterial wall by an LP×TG motif. The roles of these genes in the invasiveness of the GAS M/*emm*3 genotype are under investigation.

### Nucleotide sequence accession number.

The *S. pyogenes* strain STAB902 genome sequence has been deposited in the NCBI database under the accession no. CP007041. The version described in this paper is the first version.
